# Redundancy between nucleases required for homologous recombination promotes PARP inhibitor resistance in the eukaryotic model organism *Dictyostelium*

**DOI:** 10.1093/nar/gkx639

**Published:** 2017-07-24

**Authors:** Anna-Lena Kolb, Alasdair R. Gunn, Nicholas D. Lakin

**Affiliations:** Department of Biochemistry, University of Oxford, South Parks Road, Oxford, OX1 3QU, UK

## Abstract

ADP-ribosyltransferases promote repair of DNA single strand breaks and disruption of this pathway by Poly(ADP-ribose) polymerase (PARP) inhibitors (PARPi) is toxic to cells with defects in homologous recombination (HR). Here, we show that this relationship is conserved in the simple eukaryote *Dictyostelium* and exploit this organism to define mechanisms that drive resistance of the HR-deficient cells to PARPi. *Dictyostelium* cells disrupted in *exonuclease I*, a critical factor for HR, are sensitive to PARPi. Deletion of *exo1* prevents the accumulation of Rad51 in chromatin induced by PARPi, resulting in DNA damage being channelled through repair by non-homologous end-joining (NHEJ). Inactivation of NHEJ supresses the sensitivity of *exo1^−^* cells to PARPi, indicating this pathway drives synthetic lethality and that in its absence alternative repair mechanisms promote cell survival. This resistance is independent of alternate-NHEJ and is instead achieved by re-activation of HR. Moreover, inhibitors of Mre11 restore sensitivity of *dnapkcs^−^exo1^−^* cells to PARPi, indicating redundancy between nucleases that initiate HR can drive PARPi resistance. These data inform on mechanism of PARPi resistance in HR-deficient cells and present *Dictyostelium* as a convenient genetic model to characterize these pathways.

## INTRODUCTION

Cellular DNA is continually being damaged either by agents generated as a consequence of cellular metabolism or through exposure to genotoxic agents (1). If left unrepaired, these lesions contribute to genome instability and mutagenesis. As such cells have evolved a network of pathways termed the DNA damage response (DDR) that detect and signal DNA damage to restore genome integrity through DNA repair. The importance of the DDR is underscored by the findings that defects in these pathways results in chromosomal instability, congenital abnormalities, immunological deficiencies, neurodegeneration and cancer predisposition (2).

ADP-ribosyltransferases (ARTs), or Poly(ADP-ribose) polymerases (PARPs), catalyse the addition of single or poly-ADP ribose moieties onto target proteins (3,4). Of the 17 genes containing predicted ART catalytic domains in humans (5), several detect and signal DNA damage to facilitate repair (3,4). PARP1, the founder member of this family, signals DNA single strand breaks (SSBs) generated directly through oxidative DNA damage, or as a consequence of processing damage during base excision repair (BER) (6). Upon binding DNA SSBs, PARP1 becomes activated and ADP-ribosylates substrates at DNA lesions. This, in turn, promotes the recruitment of XRCC1 to DNA lesions that acts as a scaffold to assemble DNA processing and repair factors at damage sites (7–10). PARP2 also contributes to the repair of SSBs, particularly those generated as a consequence of BER (11,12), although its relationship to PARP1 in this process remains unclear. PARP1 is additionally required to repair other varieties of DNA damage. For example, its depletion compromises restart of stalled and/or damaged replication forks (13–15), in addition to alternative non-homologous end-joining (alt-NEHJ), a DNA double strand break (DSB) repair pathway activated in the absence of core NHEJ (c-NHEJ) factors (16). Whilst PARP1 has also been implicated in c-NHEJ (15,17), PARP3 mono-ADP-ribosylates target proteins in response to DSBs to promote the accumulation of NHEJ factors at damage sites (18–20). The DNA damage responsive ART family has been further expanded in recent years by the identification that PARP14 and PARP10 combat DNA replication stress through promoting HR and translesion DNA synthesis, respectively, at stalled replication forks (21,22).

PARP inhibitors (PARPi) are toxic to cells with defects in HR-mediated DSB repair, including cells with mutations in *BRCA1* and *BRCA2*, genes whose loss of function predisposes patients to breast and ovarian cancer (23,24). This observation provided the potential to use PARPi in a synthetic lethal strategy to specifically target HR-deficient tumours that was recently realized by the approval of Olaparib/Lynparza as a single agent therapy to treat HR-deficient ovarian tumours (25,26). This therapy works through PARPi trapping ARTs at unrepaired SSBs to elicit replication blockage, resulting in fork collapse and generation of potentially lethal DSBs (26,27). Under normal circumstances, these lesions are effectively resolved through HR-dependent repair mechanisms, events that are dependent on BRCA1 and BRCA2. However, in the absence of this pathway, DNA damage is instead channelled through mutagenic repair pathways, resulting in genome instability and cell death (25,26). Thus, PARPi are toxic to HR-defective tumour cells whilst HR-competent non-tumour cells are viable.

Whilst the basis of synthetic lethality between ART inhibition and HR-deficiency is becoming increasingly well defined, how HR-deficient cells become refractory to these agents through activation of compensatory repair mechanisms or other pathways remains unclear. One way to decipher these complex interactions is to exploit genetically tractable model organisms to understand these relationships and extend these concepts to humans. However, this approach is hampered by the absence or limited conservation of ARTs in commonly used invertebrate models exploited to study DNA repair. Recently, however, we and others identified that vertebrate DNA repair pathway components are conserved in the genetically tractable eukaryote *Dictyostelium discoideum* (28–31), including several proteins containing predicted ART catalytic domains (32). Similar to humans, two ARTs (Adprt2 and Adprt1b) are required to confer resistance to DNA SSBs (32,33). Moreover, analogous to human PARP3, we identified a third ART (Adprt1a) that responds to DNA DSBs to facilitate NHEJ (32,33). ADP–ribose interaction domains are conserved in *Dictyostelium* and are required to assemble repair factors at DNA lesions, indicating the mechanistic basis of how ARTs regulate DNA repair is also conserved in this organism (33–35). These observations, in addition to the genetic tractability of *Dictyostelium*, make it an attractive model to assess the role of ARTs in a variety of pathways, including DNA repair, in addition to the genetic interactions that influence how ART dysfunction impacts on cell viability of HR-deficient cells.

Here, we extend this work to assess pathways that drive synthetic lethality between ART inhibition and defects in HR. We identify differential inhibition of SSB and DSB-responsive *Dictyostelium* ARTs using currently available PARPi. Importantly, PARPi are toxic to *Dictyostelium* cells disrupted in the *exonuclease I* gene (*exo1*), a nuclease that initiates HR through DNA end resection (36), illustrating synthetic lethality between ART inhibition and HR-deficiency is conserved in this organism. We additionally find that disruption of the NHEJ pathway suppresses sensitivity of *exo1^−^* cells to PARPi, indicating that alternate repair mechanisms are engaged to promote cell viability. Whilst components of the alt-NHEJ pathway are dispensable in this respect, resistance is driven by restoration of HR, a process that is dependent on the Mre11 nuclease. Together, these data define the mechanisms of synthetic lethality between ART inhibition and HR-deficiency and provide insights into how resistance to these agents can be overcome.

## MATERIALS AND METHODS

### Cell culture and strain generation

All strains were grown axenically using standard procedures or in association with *Klebsiella aerogenes* on SM agar. Generation of *dnapkcs^−^* strains was previously described (29). To generate the *exo1* disruption strain, DNA fragments upstream (nucleotides −1014 to −46, primers: 5′-AGGTACCTCTA GAAAAGGTAAATTAATCATTG-3′ and 5′-CAAAGCTTCCTCCACTCCTACCTATCTATTCACC-3′) and downstream (nucleotides 2635–3441, primers: 5′-AACTGCAGCCCAAGTAGTATCGGTGATGAC-3′; 5′-CCGGATC CCACGTGGTGCACCTTCACTTTTTGGTCC-3′) of the *exo1* start codon were generated by polymerase chain reaction (PCR) from Ax2 genomic DNA (37). These fragments were cloned on either side of the floxed blasticidin resistance cassette contained within the pLPBLP plasmid (38) using KpnI and PmlI. A similar procedure was used to disrupt the *polq* gene. Thereby, DNA fragments upstream (nucleotides −714 to −4) and downstream (nucleotides 4336–5072) of the *polq* start codon were amplified using the following primers: 5′-GGGGTACCCAGTTCTCAAGTAATTTCAAAAGAG-3′ and 5′-CCGTCGACCCC CATTTCTTTTTTATCTTTATT-3′ as well as 5′-GGCCGCGGCTAAAGAACCCCAATTAGTG CC-3′ and 5′-GGGGATCCGTATCACTTAGAAATTCTTTTACTTGTGC-3′. The fragments were cloned on either side of the floxed blasticidin resistance cassette into pLPBLP using KpnI and SacII. For the *lig3* disruption strain DNA fragments upstream (nucleotides −753 to 49, primers 5′-TGGTATGGATCCAGTTACAAGAGG-3′ and 5′-CTGCAGCACATAATTTAT ACATTGAGTAAAATGATCCTG-3′) and downstream (nucleotides 2187–3112, primers: 5′-ATCGATAACAGCAACAACAAGAACCATC-3′ and 5′-GGTACCGAACCAAAACATTGA TCAACCC-3′) of the *lig3* start codon were cloned into pLPBLP using BamHI and PvuI, flanking the floxed blasticidin resistant cassette. Transfection occurred in exponentially growing *Dictyostelium* cells using standard procedures, 10 μg/ml blasticidin was added as selection the following day. Disrupted strains were isolated using standard procedures and confirmed by PCR and either Southern blotting or RT-PCR (Supplementary Figures S2–4). The following primers were used for screening of disruption strains: P1 5′- GATGGTGATAGTAATGGTGATGG-3′, P2 5′-CTTCTTTTTCATCCTCGTAGTCAC-3′, P3 5′- GCAAGGTGATATGCGTTACATAG-3′, P4 5′- ATGCTATACGAAGTTATCCGTGG-3′, P5 5′- GAAGTTATCATATGCCGCATGG-3′, P6 5′- TGTTGTTGTTGCTACAGCTATTC-3′, P7 5′- AATGTCACCTATAAAATCCAATTG-3′, P8 5′- GACCAACTATTTTCGTTTTAAAGG-3′, P9 5′-TCTCTCCAATCAAAAAGGTAAAGT-3′, P10 5′- GGTTATTTTTTGGTTGTTGAGA ATAAGTA-3′, P11 5′-TGATAATGATAATGGTGATGGCG-3′, P12 5′-CACCATTAACAATTG TCACCTCAG-3′, P13 5′-GGTATTGGTAGAATTGGTATTG TAGGTTG-3′, P14 5′-AAACTCG AGGTAGAAAAGTTTATAATTTTTTACATCTAAAAGATCACTC-3′. Prior to the generation of double and triple disruption strains the blasticidin-resistance cassette was removed from the relevant strains by transformation with plasmid pDEX-NLS-cre to express Cre recombinase. Blasticidin-sensitive clones were identified as previously described (38). Transfection of vectors containing the Myc-Ku70 or Flag-Rad51 occurred alongside the helper plasmid pREP using standard protocols, 10 μg/ml G418 was added as selection the following day.

### DNA damage sensitivity assays

Exponentially growing *Dictyostelium* cells were collected, resuspended to a density of 1 × 10^6^ cells/ml in HL5, and separated into 1 ml aliquots. Cells were exposed to the indicated concentrations of DNA damaging agent phleomycin (Sigma) or mock-treated and incubated at 100 rpm for 1 h whilst shaking. Afterwards, cells were diluted to 1 × 10^4^ cells/ml in KK2 and replicates of 250 cells were plated on 140 mm SM agar plates in association with *K. aerogenes*. Survival was assessed by observing *Dictyostelium* plaque formation after 3–7 days.

### Luminescence cell viability assay

Exponentially growing *Dictyostelium* cells were diluted to 2 × 10^3^ cells/ml in HL5 and 50 μl, containing 100 cells, were transferred as replicates in a 96-well plate. Cells were exposed to the indicated concentrations of mirin or ART inhibitor, such as Benzamide and NU1025 or treated with the respective carrier (100% Ethanol for Benzamide; DMSO for NU1025 and mirin). Cells were incubated for 5 days in the dark. Afterwards, the media was replaced by a 1:1 dilution of *CellTiter-Glo*^®^ reagent (Promega) in HL5 and incubated for 30 min whilst shaking. The *CellTiter-Glo*^®^ reagent induces cell lysis and generates a luminescent signal proportional to the amount of present adenosine triphosphate (ATP). The ATP level is directly proportional to the number of viable cells in the culture. Luminescence was measured using a platereader (Pherastar). After averaging the luminescence signal of the two replicates, the log2 fold change was computed by subtracting the base 2 logarithm of the carrier treated sample from the base 2 logarithm drug-treated sample.

### Chromatin extraction

Exponentially growing *Dictyostelium* cells were diluted to 1 × 10^6^ cells/ml in HL5 if treated with NU1025, and to 5 × 10^6^ cells/ml in HL5 when treated with Phleomycin. Chromatin enriched samples and whole cell extracts were prepared as previously described (33). Analysis of extracts was performed by sodium dodecyl sulphate-polyacrylamide gel electrophoresis (SDS-PAGE) and western blotting with the following primary antibodies: anti-Myc (1:1000; Santa Cruz Biotechnology), anti-H3 (1:2000; Abcam), anti-γH2AX (1:1000; Abcam), anti-actin (1:1000; Santa Cruz Biotechnology) and anti-Flag (1:5000; Sigma–Aldrich).

### HR-efficiency assays

HR-efficiency assays were performed as previously described (37). Briefly, The *cdk8* knockout construct (39) was digested with KpnI and NotI to obtain DNA fragments homologous to the *Dictyostelium cdk8* genomic locus which flank the blasticidin resistance cassette. The fragments were purified using the PCR purification kit (Qiagen) and exponentially growing *Dictyostelium* cells were transfected using standard protocols. Cells were grown for 14 days in 96-well plates with blasticidin (10 μg/ml) as selection. Clonal suspensions of blasticidin-resistant transformants were spotted onto SM agar containing a lawn of *K. aerogenes*. Plaques were grown for 5–7 days until they were large enough for phenotypic analysis. Targeted disruption of the *cdk8* genomic locus is indicated by an aggregation-deficient phenotype. Some aggregation-proficient and -deficient colonies were selected, and the genomic DNA was analysed using PCR to confirm targeted or random integration.

### ADP-ribosylation assays

Recombinant His-tagged Adprt1a or Adprt2 were expressed and purified from bacteria cells using standard protocols. A reaction containing 75 μM NAD^+^, 100 nM ^32^P-NAD^+^ (Hartmann Analytic), 50 mM Tris–HCl (pH8), 2 mM MgCl_2_ and 5 μg/ml sheared salmon sperm DNA was assembled that included the indicated concentration of the relevant ART inhibitor. One micromolar of His-tagged Adprt1a or Adprt2 were then added to the mixture to start the reaction. The reaction was incubated for 30 min room temperature and terminated by addition of 1 × SDS buffer and 1 mM Dithiothreitol (DTT) and boiling for 10 min. The reaction mixture was subjected to 8% SDS-PAGE and ADP-ribosylated proteins detected using X-ray film and Phosphorimager analysis.

### Clonogenic assay to assay synthetic lethality between PARP inhibition and HR-deficiency

Exponentially growing *Dictyostelium* cells were diluted to 2.5 × 10^3^ cells/ml in HL5 and 100 μl were transferred into a 14-cm dish. The relevant PARPi concentration was added and the plates were incubated for 9–14 days at 22°C in the dark. When using Benzamide the media was refreshed every second day to ensure PARPi activity. Afterwards, the plates were washed twice with ice cold phosphate-buffered saline followed by fixation with ice-cold Methanol for 10 min. Colonies were stained using 0.5% Crystal Violet solution. After 10 min the plates were carefully washed with ddH_2_O and dried at room temperature.

## RESULTS

### Differential inhibition of *Dictyostelium* Adprt1a and Adprt2 by ART inhibitors

**Figure 1.**
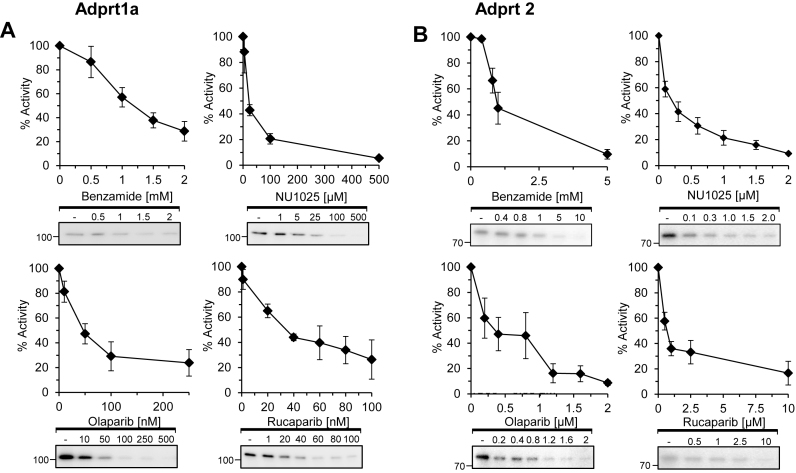
Differential inhibition of *Dictyostelium* Adprt1a and Adprt2 by ART inhibitors *in vitro*. (**A** and **B**) Recombinant His- tagged Adprt1a (A) or Adprt2 (B) were employed in ADP-ribosylation assays using ^32^P-labelled NAD+ in the absence or presence of increasing concentrations of ART inhibitors as indicated. Following SDS-PAGE, ADP-ribosylated proteins were detected and quantified by phosphorimager analysis. Error bars represent the standard error of the mean (SEM) from three independent experiments.

As an initial step to study ART inhibitors in *Dictyostelium*, we screened several of these agents to establish their efficacy for inhibiting the activity of SSB or DSB-responsive ARTs *in vitro*. Whilst the ART Adprt2 is required for tolerance of *Dictyostelium* cells to agents that induce DNA SSBs, Adprt1a signals DNA DSBs to promote NHEJ (33). We therefore focussed our analysis on these two ARTs. Human DNA damage responsive ARTs are activated by DNA strand breaks *in vitro* to undergo auto-ADP-ribosylation (40). Therefore, we expressed and purified His-tagged Adprt2 and Adprt1a from bacteria and employed these enzymes in ADP-ribosylation assays to assess how their auto-ribosylation activity is affected by a variety of PARPi. We observe an ADP-ribosylated species that co-migrates with recombinant Adprt1a or Adprt2 in our assays (Figure 1A and B), indicating that similar to human ARTs, these enzymes auto-ADP-ribosylate *in vitro*. Benzamide and NU1025 are both able to inhibit nuclear ADP-ribosylation in response to DNA SSBs (33). Consistent with this observation, we observe that these agents inhibit auto-ribosylation of the SSB-responsive ART Adprt2 (Figure 1B). Whilst similar concentrations of benzamide are required to inhibit Adprt1a and Adprt2, NU1025 inhibits Adprt2 with greater efficiency than Adprt1a (Figure 1). In contrast, olaparib and rucaparib are more effective at inhibiting Adprt1a than Adprt2. Taken together, these data illustrate differential abilities of PARPi to target *Dictyostelium* DNA damage responsive ARTs *in vitro*, and that NU1025 is the most effective PARPi to target the SSB-responsive ART Adprt2.

### Synthetic lethality between ART inhibition and HR-deficiency is conserved in *Dictyostelium*

**Figure 2.**
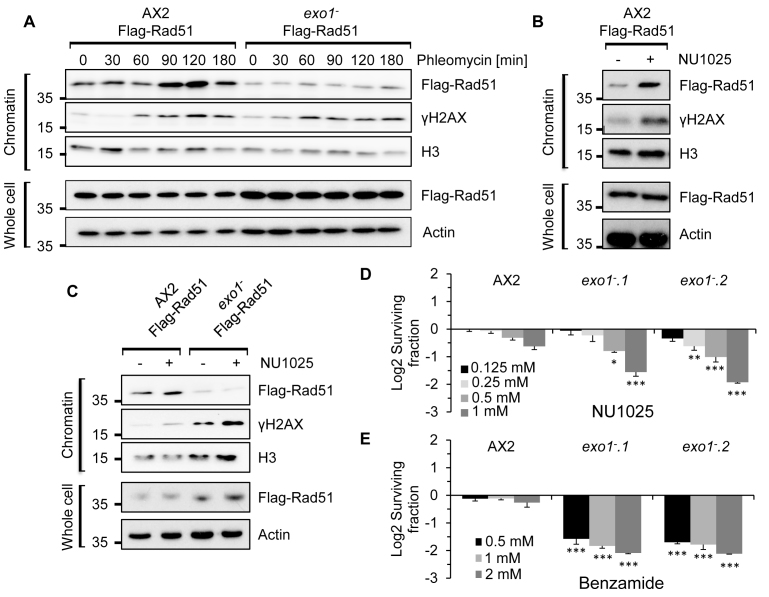
Synthetic lethality between HR-deficiency and ART inhibition is conserved in *Dictyostelium*. (**A**) Ax2 cells and *exo1^−^* cells expressing Flag-Rad51 were left untreated or exposed to 300 μg/ml of phleomycin. Chromatin and whole cell extracts were prepared after the indicated times and western blotting performed with the indicated antibodies. (**B** and **C**) Ax2 cells or *exo1^−^* cells expressing Rad51-Flag were left untreated or exposed to 1 mM NU1025 as indicated. Chromatin and whole cell extracts were prepared and western blotting performed using the indicated antibodies. (**D** and **E**) Ax2 cells and two independent *exo1^−^* strains were incubated with the indicated concentrations of NU1025 (D) or benzamide (E). Cell survival was assessed after 5 days using *CellTiter-Glo*^®^ (Promega). Cell viability is represented as log2 fold changes between untreated cells and those exposed to PARPi. Error Bars represent the standard error of the mean (SEM) from three independent experiments. Statistical significance was calculated between the *exo1^−^* strains and the Ax2 strain treated with the same PARPi concentration by two-sided student’s *t*-test *P ≤ 0.05, **P ≤ 0.01 and ***P ≤ 0.001.

Having assessed the efficacy of several PARPi *in vitro*, we next wished to establish whether any of these agents are synthetic lethal with HR-deficiency in *Dictyostelium*. To achieve this, we exploited a strain disrupted in *exo1*, a critical factor required to initiate HR in a variety of organisms, including *Dictyostelium* (37). Exo1 resects DNA DSBs to produce single stranded DNA that is recognized by Rad51 to initiate the strand invasion step of HR (36). To assess whether *Dictyostelium* Exo1 was similarly required for loading of Rad51 at sites of DNA DSBs, we expressed recombinant Flag-tagged Rad51 (Flag-Rad51) in parental Ax2 cells or the *exo1^−^* strain and assessed its ability to be enriched in chromatin following induction of DNA DSBs by the radiomimetic agent phleomycin. Whilst Flag-Rad51 is effectively enriched in chromatin in response to DSBs in parental Ax2 cells, this is compromised in the *exo1^−^* strain (Figure 2A), supporting a role for Exo1 in initiation of HR by promoting assembly of Rad51 at DNA DSBs. The current dogma dictates that disruption of SSB repair by PARPi results in increased DNA damage that is subsequently channelled through HR. Given NU1025 is the most effective PARPi that targets the *Dictyostelium* SSB-responsive ART Adprt2 (Figure 1), we tested whether this agent induces DNA damage and Rad51 engagement in *Dictyostelium* cells and whether this is dependent on Exo1. Consistent with NU1025 inducing DNA DSBs in parental Ax2 cells, elevated levels of H2AX phosphorylation (γH2AX) are apparent when these cells are incubated with this PARPi and this is reflected in the accumulation of Flag-Rad51 in chromatin (Figure 2B and C). Strikingly, whilst NU1025 induces increased levels of γH2AX in *exo1^−^* relative to Ax2 cells, enrichment of Rad51 following NU1025 exposure is reduced in the absence of Exo1, indicating that HR is unable to be engaged in response to ART inhibition in this strain (Figure 2C). We next assessed whether the inability to engage HR in the presence of NU1025 is reflected in toxicity of these agents to the *exo1^−^* strain and whether this extends to other PARPi. Whilst Ax2 cells could tolerate the presence of NU1025 in media, this agent is toxic to *exo1^−^* cells (Figure 2D and Supplementary Figure S1). Similarly, benzamide was also toxic to *exo1^−^* cells relative to parental Ax2 controls, indicating this observation is not restricted to NU1025 (Figure 2E and Supplementary Figure S1). Taken together these data indicate that PARPi induce DNA damage is channelled through HR-dependent repair mechanisms and that synthetic lethality between ART inhibition and HR-deficiency is conserved in *Dictyostelium*.

### Disruption of NHEJ supresses the sensitivity of the HR-deficient cells to PARPi

**Figure 3.**
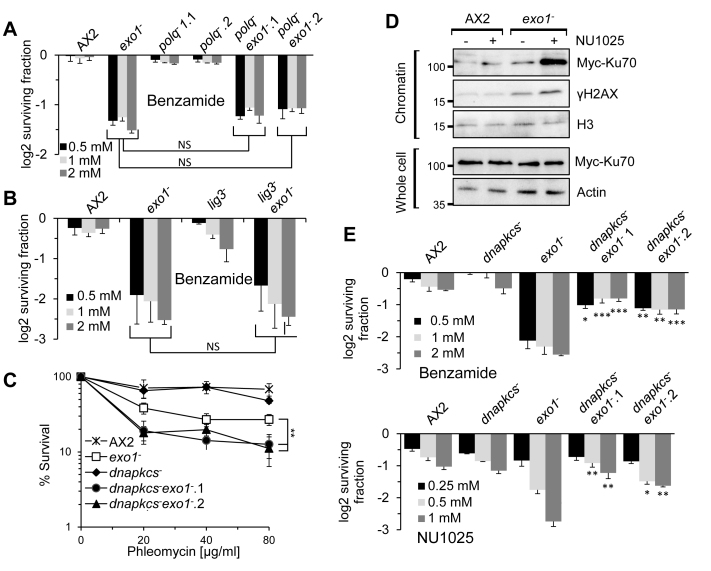
Disruption of NHEJ suppresses the sensitivity of HR-deficient strains to ART inhibitors. (**A** and **B**) The indicated strains were incubated with increasing concentrations of Benzamide. Cell viability was assessed after 5 days using *CellTiter-Glo*^®^ (Promega). Cell viability is represented as log2 fold changes between untreated and treated samples. Error bars represent the SEM from three independent experiments. *P*-values were calculated by two-sided student’s *t*-test between the double knockout strains (*polq^−^exo1*^−^ for A, *lig3^−^exo1*^−^ for B) and the *exo1^−^* strain, treated with the same PARPi concentration, NS *P* > 0.05. (**C**) Ax2, *exo1^−^, dnapkcs^−^* and two independent *dnapkcs^−^exo1^−^* strains were assessed for survival after treatment with the indicated concentrations of phleomycin. Error bars represent the SEM from three independent experiments. Statistical significance was determined for the *dnapkcs^−^exo1^−^* strains compared to the *exo1^−^* strain by two-way Annova, *P ≤ 0.05, **P ≤ 0.01. (**D**) Ax2 and *exo1^−^* cells expressing Myc-Ku70 were left untreated or exposed to 1 mM NU1025. Chromatin and whole cell extracts were prepared from cells and western blotting performed with the indicated antibodies. (**E**) Ax2, *exo1^−^, dnapkcs^−^* and two independent *dnapkcs^−^exo1^−^* cells were treated with the indicated concentrations of benzamide (upper panel) or NU1025 (lower panel). Cell survival was assessed after 5 days using *CellTiter-Glo*^®^ (Promega). Cell viability is represented as log2 fold changes between untreated cells and those exposed to PARPi. Error Bars represent the SEM from three independent experiments. Statistical significance was determined between the *dnapkcs^−^exo1^−^* and the *exo1^−^* strains, exposed to the respective PARPi concentration by two-sided student’s *t*-test, *P ≤ 0.05, **P ≤ 0.01 and *** *P* ≤ 0.001.

The elevated levels of DNA damage induced by NU1025 in *exo1^−^* cells suggests that DSB repair pathways other than HR may be engaged in these cells that could either suppress or exacerbate the sensitivity of these cells to PARPi. In addition to HR, DSBs can be repaired by alt-NHEJ and components of this pathway, including LigIII and PolQ, are conserved in *Dictyostelium* (www.dictybase.org) (32). Recently, alt-NHEJ has been suggested to compete with HR, particularly in the context of resolving replication stress (41,42), making it an attractive alternative pathway to engage in the absence of HR at stalled/damaged replication forks induced by PARPi. Therefore, we disrupted *ligIII* or *polq* alone or in combination with *exo1* and assessed the ability of the resulting strains to survive exposure to NU1025 and benzamide. Consistent with previous observations (Figure 2D and E), benzamide (Figure 3A and B) and NU1025 (Supplementary Figure S5A and B) are toxic to *exo1^−^* cells relative to parental Ax2. However, disruption of *ligIII* or *polq* did not dramatically impact on cell viability following incubation with PARPi either in the absence or presence of *exo1* (Figure 3A and B; Supplementary Figure S5A and B), indicating these genes do not impact on the ability of cells to tolerate DNA damage induced by PARPi.

We next considered whether c-NHEJ is engaged in the absence of Exo1 and how this impacts on the ability of cells to tolerate PARPi. Restriction enzyme mediated integration of DNA into the genome of *Dictyostelium* and resumption of cell growth following exposure to DNA DSBs is defective in NHEJ mutants (37,43), indicating an active NHEJ pathway in vegetative *Dictyostelium* cells. However, whilst *exo1^−^* cells are sensitive to DNA DSBs (37), vegetative NHEJ mutants do not exhibit a significant increase in cell death relative to parental control cells (29,37), suggesting HR is the primary pathway employed to promote resistance to DSBs at this stage of the *Dictyostelium* life cycle. To assess whether cells become reliant on NHEJ when HR is dysfunctional, we disrupted the *exo1* gene in a *dnapkcs*^−^ background and compared the ability of these cells to tolerate DSBs relative to strains disrupted in either gene alone. Similar to previous reports (29) and in contrast to *exo1^−^* cells, vegetative *dnapkcs*^−^ cells do not exhibit a significant sensitivity to the DSB-inducing agent phleomycin. However, disruption of *dnapkcs* further sensitizes *exo1^−^* cells to DSBs (Figure 3C), indicating that in the absence of HR, c-NHEJ is employed to allow cells to tolerate DSBs. This raises the possibility that the c-NHEJ pathway may also be engaged in *exo1^−^* cells in response to PARPi. Consistent with this hypothesis, PARPi induce an enrichment of Ku70 in chromatin fractions in *exo1^−^* cells above that observed in parental Ax2 cells, suggesting DNA damage is channelled through the c-NHEJ pathway in this context (Figure 3D). Moreover, we observe that disruption of the *dnapkcs* gene in *exo1^−^* cells suppresses the sensitivity of this strain to PARPi (Figure 3E), indicating that in the absence of HR PARPi induce NHEJ and that this is toxic to cells. Intriguingly, we also see a mild increase in the tolerance of *dnapkcs^−^* cells to PARPi, suggesting that disruption of the NHEJ pathway may also impact on the ability of cells to tolerate these agents in an HR proficient background.

### PARPi resistance is driven by reactivation of HR

**Figure 4.**
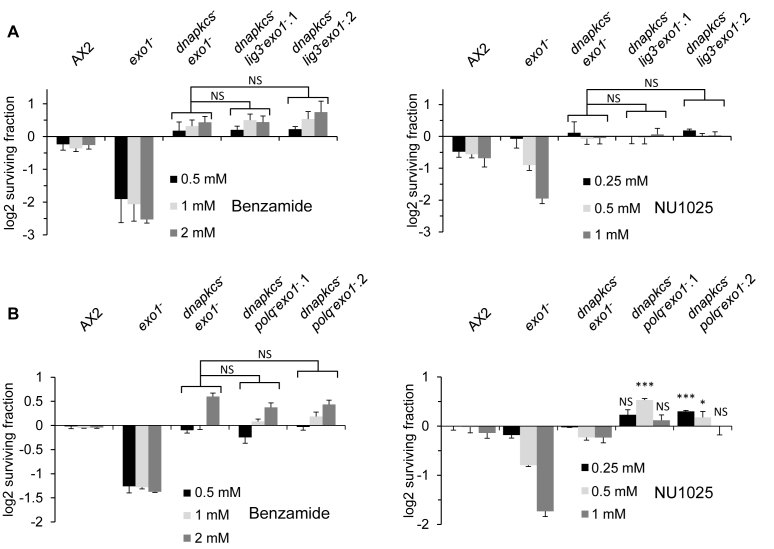
Survival of *dnapkcs^−^exo1^−^* in the presence of PARPi is independent of the alt-NHEJ pathway. (**A**) Ax2, *exo1^−^, dnapkcs^−^exo1^−^* and two independent *dnapkcs^−^lig3^−^exo1^−^* cells were treated with the indicated concentrations of benzamide (left panel) or NU1025 (right panel). Cell viability was assessed after 5 days using *CellTiter-Glo*^®^ (Promega). Cell viability is represented as log2 fold changes between untreated cells and those exposed to PARPi. Error Bars represent the SEM from three independent experiments. *P*-values were determined for the *dnapkcs^−^lig3^−^exo1^−^* strains compared to the *dnapkcs^−^exo1^−^* strain exposed to the same PARPi concentration using two-sided student’s *t*-test, NS *P* > 0.05. (**B**) Ax2, *exo1^−^, dnapkcs^−^exo1^−^* and two independent *dnapkcs^−^polq^−^exo1^−^* cells were treated with the indicated concentrations of benzamide (left panel) or NU1025 (right panel). Cell viability was assessed as described in (A). *P*-values were determined for the *dnapkcs^−^polq^−^exo1^−^* strains compared to the *dnapkcs^−^exo1^−^* strain exposed to the same PARPi concentration using two-sided student’s *t*-test, NS *P* > 0.5, *P ≤ 0.05, **P ≤ 0.01 and ***P ≤ 0.001.

The suppression of PARPi toxicity to HR-deficient cells by disruption of the NHEJ pathway indicates other repair mechanisms are engaged in this context to promote cell viability. Given alt-NHEJ is employed to repair canonical DSBs in the absence of c-NHEJ (16), we first tested whether LigIII or PolQ is responsible for allowing *dnapkcs*^−^*exo1*^−^ cells to tolerate PARPi. Accordingly, we generated *dnapkcs^−^ligIII^−^exo1^−^* or *dnapkcs^−^polq^−^exo1^−^* strains and tested whether this could re-sensitize the *dnapkcs*^−^*exo1*^−^ strain to PARPi. However, neither the *dnapkcs^−^ligIII^−^exo1^−^* or *dnapkcs^−^polq^−^exo1^−^* strains exhibited increased sensitivity following exposure to benzamide or NU1025 relative to *dnapkcs^−^exo1^−^* cells (Figure 4A and B), indicating that LigIII or PolQ do not play a significant role in allowing HR/NHEJ-deficient cells to tolerate PARPi.

**Figure 5.**
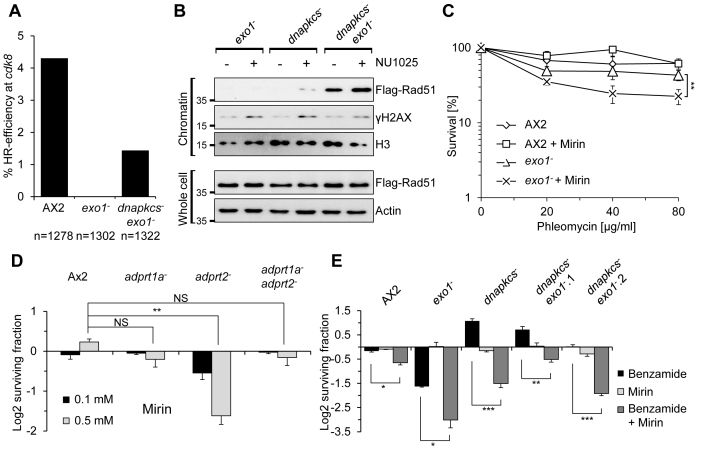
PARPi resistance is due to restoration of HR. (**A**) Ax2, *exo1^−^* and *dnapkcs^−^exo1^−^* cells were assessed for HR efficiency by measuring targeted integration of the hygromycin cassette at the *cdk8* locus. Multiple transfections were performed and drug-resistant clones analysed for targeting *cdk8* by PCR analysis. The percentage of targeted HR efficiency at the *cdk8* locus is calculated as the proportion of aggregate-null colonies against the total number of colonies analysed. The *n* number represents the total number of clones analysed. (**B**) The indicated strains, all of which are expressing Flag-Rad51, were left untreated or exposed to 1 mM NU1025. Chromatin and whole cell extracts were prepared and western blotting performed using the indicated antibodies. (**C**) Ax2 and *exo1^−^* cell were pre-incubated for 30 min with or without 0.5 mM mirin prior to being left untreated or exposed to the indicated concentrations of phleomycin. Cell survival was assessed as described in ‘Materials and Methods’ section. Error bars represent the SEM from three independent experiments. Statistical significance was determined for the *exo1^−^* strain exposed to mirin compared to the mock treated *exo1^−^* strain by two-way Annova, *P ≤ 0.05, **P ≤ 0.01. (**D**) Ax2, *adprt1a^−^, adprt2^−^* and *adprt1a^−^adprt2^−^* strains were treated with the indicated mirin concentration. Cell viability was assessed after 5 days using *CellTiter-Glo*^®^ (Promega). Cell viability is represented as log2 fold change between untreated cells and those exposed to PARPi. Error bars represent the SEM from three independent experiments. Statistical significance was calculated for the disruption strains compared to the Ax2 strain exposed to the same mirin concentration using two-sided student’s *t*-test, NS *P* > 0.05, *P ≤ 0.05, **P ≤ 0.01. (**E**) Ax2, *exo1^−^, dnapkcs^−^* and two independent *dnapkcs^−^exo1^−^* strains were treated with 0.5 mM mirin and/or 2 mM benzamide as indicated. Cell viability was assessed after 5 days using *CellTiter-Glo*^®^ (Promega). Cell viability is represented as log2 fold change between untreated cells and those exposed to PARPi. Error Bars represent the SEM from three independent experiments. Statistical significance was calculated for the *dnapkcs^−^exo1^−^* strains exposed to mirin and PARPi compared to the *dnapkcs^−^exo1^−^* strains treated only with PARPi using two-sided student’s *t*-test,*P ≤ 0.05, **P ≤ 0.01 and ***P ≤ 0.001.

We next assessed whether re-activation of HR allows *dnapkcs^−^exo1^−^* cells to tolerate PARPi. Initially we established the ability of *dnapkcs^−^exo1^−^* strains to perform HR by exploiting an assay that measures efficiency of this repair mechanism by quantifying HR-mediated gene replacement at the *cdk8* locus (37). As expected, *exo1^−^* cells are unable to perform HR relative to parental Ax2 cells (Figure 5A). However, disruption of *dnapkcs* in the *exo1^−^* strain restores the ability of these cells to perform targeted-HR (Figure 5A). We also assessed whether restoration of HR is reflected in the ability of *dnapkcs^−^exo1^−^* cells to load Rad51 onto chromatin in response to PARPi. Consistent with previous observations, we observe an inability of Flag-Rad51 to be enriched in chromatin fractions prepared from *exo1^−^* cells following exposure to NU1025. Strikingly, however, we observe an increase in the levels of Flag-Rad51 in chromatin fractions prepared from *dnapkcs^−^exo1^−^* cells and this is further increased when cells are exposed to PARPi, supporting our conclusion that HR is reactivated in these cells (Figure 5B). Therefore, disruption of NHEJ in *exo1^−^* cells can restore HR-mediated repair of DNA damage induced by PARPi, providing an explanation for why these cells are refractory to PARPi.

Given DNA-end resection is a pre-requisite to engage Rad51 at sites of DNA damage, the ability to load Rad51 onto chromatin in the *dnapkcs^−^exo1^−^* strain suggests that other nucleases are able to substitute for loss of Exo1 and initiate the HR pathway. Therefore, we tested whether other nucleases required for DNA end-resection are redundant with Exo1 and if disruption of these activities could re-sensitize the *dnapkcs^−^exo1^−^* strain to PARPi. Whilst yeast Exo1 is required for extensive resection of canonical DSBs induced by endonucleases, in its absence limited resection can still be achieved by Mre11 (44–47). Therefore, we considered whether Mre11 is driving the resistance of *dnapkcs^−^exo1^−^* cells to PARPi. With the exception of *exo1*, we and others have been unable to delete core HR genes, suggesting that their disruption is lethal (29,31). Therefore, to assess the relationship between Exo1 and Mre11 in PARPi resistance, we employed the Mre11 inhibitor mirin in our experiments (48). Initially, we assessed whether mirin could further sensitize the *exo1^−^* strain to DSBs. Whilst this agent does not sensitize Ax2 cells to phleomycin, it does induce further sensitization of *exo1^−^* cells to DSBs (Figure 5C), indicating redundancy between these nucleases with respect to tolerance to this variety of DNA damage. To probe the requirement for ARTs in synthetic lethality with HR deficiency, we also assessed the impact of this compound on cell viability in different ART-defective backgrounds. Whilst *adprt1a^−^* cells were able to tolerate exposure to mirin similar to parental Ax2 cells, consistent with the synthetic lethal relationship between PARP and HR dysfunction, the SSBR defective *adprt2^−^* strain is sensitive to mirin (Figure 5D). Strikingly, disruption of *adprt1a* suppressed this phenotype of *adprt2^−^* cells, indicating that in the absence of Adprt2, Apdrt1a is toxic in combination with Mre11 inhibition. Moreover, whilst mirin slightly decreased the ability of *exo1^−^* or parental Ax2 cells to tolerate benzamide, it more significantly re-sensitized the *dnapkcs^−^exo1^−^* strain to this agent (Figure 5E), indicating that in the absence of *exo1*, Mre11 can catalyse DNA end-resection to promote HR and tolerance of cells either to DSBs or PARPi.

## DISCUSSION

Our previous work identified that ARTs are conserved in *Dictyostelium* and contribute to repair of DNA SSBs and DSBs in a manner that is mechanistically similar to humans (32). Whilst Adprt2 is required for tolerance of cells to agents that induce DNA SSBs, Adprt1a is largely dispensable in this respect and instead regulates repair of DSBs through NHEJ by promoting interaction of Ku70 at DSBs through a PAR interaction domain situated at its C-terminus (33). Here we extend our studies of ARTs in *Dictyostelium* to illustrate that synthetic lethality between ART inhibition and HR-deficiency is similarly conserved in this organism. Moreover, we identify that HR-deficient cells can become refractory to PARPi through loss of the NHEJ pathway and importantly, that this resistance mechanism is driven by restoration of HR through redundant nucleases that promote end-resection to initiate this repair pathway.

Our data indicate that different PARPi exhibit differential inhibition of SSB and DSB-responsive ARTs *in vitro*. Whilst benzamide is equally effective at inhibiting Adprt2 and Adprt1a auto-catalytic activity *in vitro*, NU1025 preferentially targets the SSB-responsive Adprt2. The prevailing view of PARPi toxicity to HR-deficient cells is that inhibition of PARP1 and PARP2 disrupts SSB repair by trapping these ARTs at DNA breaks. This results in replication stress when ART-bound DNA lesions are encountered during DNA synthesis, creating a dependency on HR-mediated repair for replication fork recovery (26,27). Consistent with this model, we observe that benzamide and NU1025 are synthetic lethal with loss of HR (Figure 2D and E). In contrast, olaparib and rucaparib show a preferential inhibition of the DSB-responsive ART Adprt1a (Figure 1) and intriguingly, neither of these PARPi are toxic to *exo1^−^* cells (Supplementary Figure S5C and D). Whilst a number of pharmokinetic properties of *Dictyostelium* cells may influence the efficacy of these agents in our experimental system (e.g. cellular import/export, compound stability etc.), these data suggest that inhibition of Adprt1a has little impact on the ability of cells to tolerate loss of HR. In further support of this model, we observe that *adprt1a^−^* cells are able to tolerate the Mre11 inhibitor mirin, whilst this compound is toxic to *adprt2^−^* cells (Figure 5D).

The accumulation of DNA damage in *exo1^−^* cells treated with PARPi led us to consider if other DSB repair mechanisms are engaged in this context. HR is the predominant pathway that allows *Dictyostelium* cells to tolerate DNA DSBs; whilst deletion of *exo1* results in sensitivity of *Dictyostelium* cells to phleomycin during vegetative cell growth, disruption of NHEJ at this stage of the life cycle has little impact on cell viability in response to DSBs (29). Nevertheless, integration of plasmid DNA into the genome of *Dictyostelium* is dependent on Ku and DNA-PKcs (37), and NHEJ mutants are unable to resume cell growth following exposure to DSB-inducting agents (43), indicating NHEJ is competent at this stage of the *Dictyostelium* life cycle. Consistent with this hypothesis, we observe that disruption of NHEJ further sensitizes HR-deficient cells to DSBs (Figure 3C). Therefore, whilst HR is the predominant pathway to repair canonical two-sided DNA DSBs, in the absence of this pathway DSBs are channelled through NHEJ. In support of NHEJ also being engaged in response to PARPi in the absence of HR, whilst PARPi-induced Rad51 enrichment in chromatin is defective in *exo1^−^* cells, we instead see an accumulation of Ku70 in this context (Figures 2C and 3D). In stark contrast to canonical DSBs, however, disruption of the NHEJ pathway suppresses the sensitivity of *exo1^−^* cells to PARPi (Figure 3E), indicating that engagement of this pathway is toxic to cells in this context. In further support of this model, we observe that disruption of Adprt1a, the *Dictyostelium* ART that regulates NHEJ (33), supresses the sensitivity of the SSBR defective *adprt2^−^* cells to an inhibitor of the HR protein Mre11 (Figure 5D). Together, these observations indicate that NHEJ may be toxic or beneficial depending on the context of the DSB. For example, NHEJ is considered an undesirable pathway to engage for repair of DNA DSBs sustained during DNA replication and disruption of this pathway can supress the toxicity of DNA damaging agents to cells with defects in the Fanconi Anaemia pathway, a DNA repair mechanism engaged during DNA replication (49–52). Importantly, however, they also suggest that PARPi which target both NHEJ and SSB responsive ARTs may have a reduced efficacy to those which inhibit SSB repair alone. Currently available ART inhibitors are capable of binding to and inhibiting PARP1 and PARP3, human ARTs that regulate SSBR and NHEJ, respectively (19,53,54). Therefore, our data would argue for the development of PARP1-specific inhibitors with a view to increasing efficacy of these agents in therapies that target HR-deficient tumours.

The observation that *dnapkcs^−^exo1^−^* cells are able to tolerate PARPi raises the question of which repair pathway is engaged in these cells to resolve DNA damage. Recent findings indicate that alt-NHEJ competes with HR to repair DNA damage (41,42), raising the possibility that this pathway is engaged in *exo1^−^* cells to maintain cell viability in the presence of PARPi. However, we observe no impact on disrupting *ligIII* or *polq* on tolerance of *exo1^−^* cells to PARPi either in the presence or absence of NHEJ (Figures 3A–B and 4). Although we cannot formally exclude the possibility that loss of *ligIII* or *polq* does not fully disrupt the alt-NHEJ pathway, these data suggests that this repair mechanism plays little if any role in allowing HR-deficient cells to tolerate PARPi. Given alt-NHEJ is regulated by ARTs in humans (55–58), this is perhaps not surprising, and may indicate that ARTs are similarly required for the regulation of this repair pathway in *Dictyostelium*.

Our data instead indicate that disruption of the NHEJ pathway allows *exo1^−^* cells to tolerate PARPi through restoration of HR (Figure 5). Resection of DNA DSBs into single-stranded DNA intermediates is a critical point in regulating whether repair of DSBs is channelled through NHEJ or HR (59). 53BP1 and its binding partners RIF1 and REV7 compete with HR proteins such as BRCA1, protecting DNA ends from resection and channelling repair through NHEJ (60). Consistent with this being a pivotal decision point that influences the ability of HR-deficient cells to tolerate PARPi, loss of 53BP1, RIF1 or REV7 supresses HR-defects of BRCA1-deficient cells, resulting in tolerance to PARPi (51,61–64). Our data indicate that disruption of DNA-PKcs is able to restore loading of Rad51 onto chromatin in response to PARPi, suggesting that disruption of core NHEJ factors are similarly able to promote end resection to allow cells to become resistant to PARPi. This is reminiscent of the situation at canonical DSBs, where loss of Ku and other core NHEJ factors can similarly lead to unprotected DNA ends, resulting in an increased ability to resect DSBs and channel repair through HR (59,65,66). Importantly, however, our data also indicate that redundancy between nucleases required for DNA resection can drive the resistance of the HR-deficient cells to PARPi. Redundancy between Mre11, Exo1 and DNA2 in resection of DNA DSBs is observed in yeast and humans (36). Moreover, whilst yeast Exo1 is required for extensive resection of DSBs to promote NHEJ, in the absence of this factor limited resection can still be achieved by Mre11 and DNA2 (44–47). Our data illustrating that inhibition of Mre11 can restore sensitivity of *dnapkcs^−^exo1^−^* cells to PARPi, suggest that similar redundancy between these nucleases is a determining factor in tolerance to PARPi. In addition to HR, Mre11 has also been implicated in DNA resection events that initiate alt-NHEJ (16), raising the possibility that this pathway may be responsible for resistance of *dnapkcs^−^exo1^−^* cells to PARPi. However, as described above, given alt-NHEJ is dependent on ARTs, we feel it is unlikely this repair pathway will be responsible for this resistance. In further support of this hypothesis, disruption of LigIII and PolQ does not restore sensitivity of the *dnapkcs^−^exo1^−^* strain to PARPi (Figure 4). Therefore, we propose that disruption of NHEJ renders DNA damage sites generated in response to PARPi more susceptible to end resection, allowing Mre11 to process DNA ends and promote HR-mediated tolerance to PARPi.

In summary, we exploit the conservation of ARTs in *Dictyostelium* to assess the role of ADP-ribosylation in DNA repair and identify that synthetic lethality between ART inhibition and HR-deficiency is conserved in this organism. Additionally, we use this unique experimental system to examine pathways that supress this genetic relationship and identify that restoration of HR through redundant mechanism used to resect DNA DSBs promotes resistance of HR-deficient cells to PARPi. Given nucleases are drugable targets, this raises the possibility of intervention in drug resistance mechanisms to overcome HR-deficient tumours resistance to treatment with PARPi.

## Supplementary Material

Supplementary Data
