# Supplementary material for: Redundancy between nucleases required for homologous recombination promotes PARP inhibitor resistance in the eukaryotic model organism Dictyostelium
Source: Nucleic Acids Res. 2017 Jul 24;45(17):10056–67. doi: 10.1093/nar/gkx639 (PMC5622368; doi:10.1093/nar/gkx639)
Supplement: Supplementary Data [file gkx639_supp.zip › nar-01073-d-2017-File007.pdf]

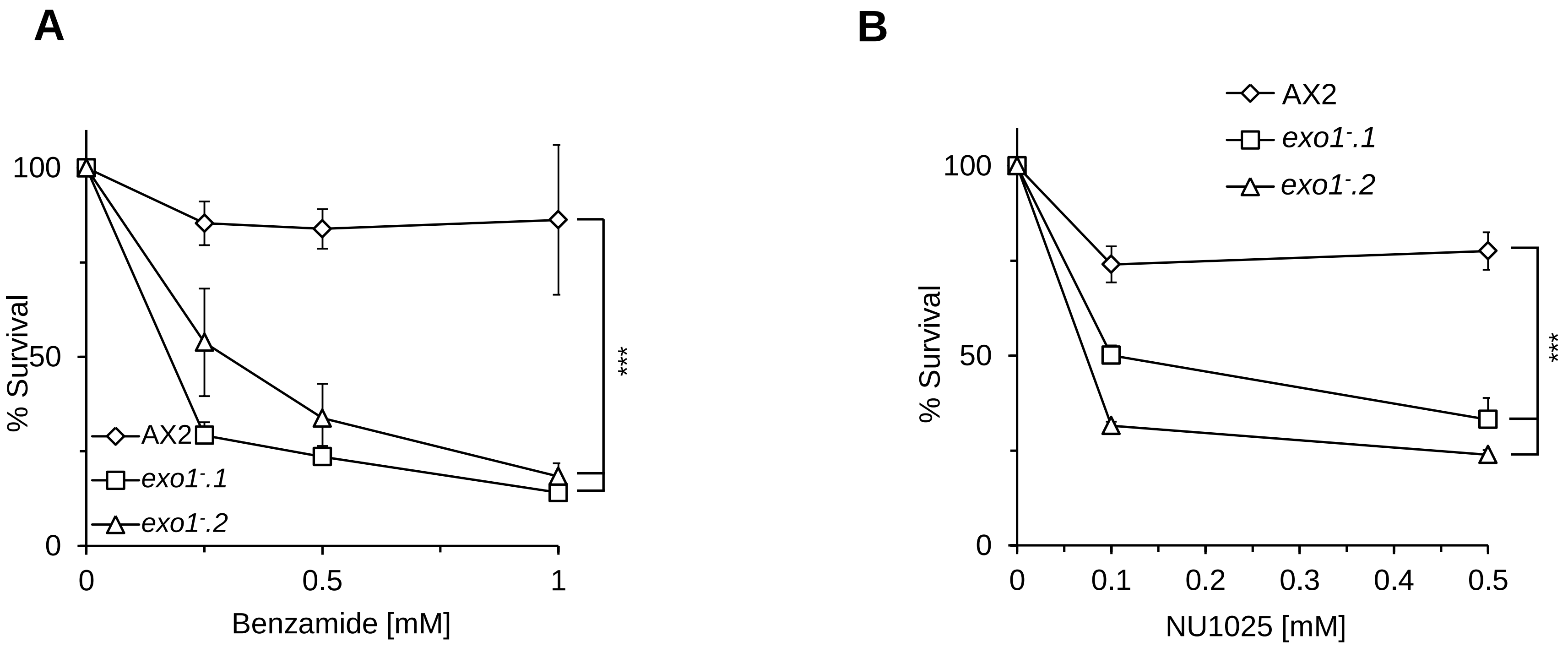

Supplementary Figure S1

A and B. Ax2 and two independent *exo1*$^{-}$ strains were incubated with the indicated PARPi concentration or mock treated. Cell survival was assessed by observing *Dictyostelium* colony formation after 9-14 days as described in the Materials and Methods. Error Bars represent the SEM from three independent experiments. Statistical significance was determined for the *exo1*$^{-}$ strain compared toAx2 strains by two-way Annova, * P≤0.05, ** P ≤ 0.01, ***P ≤ 0.001.

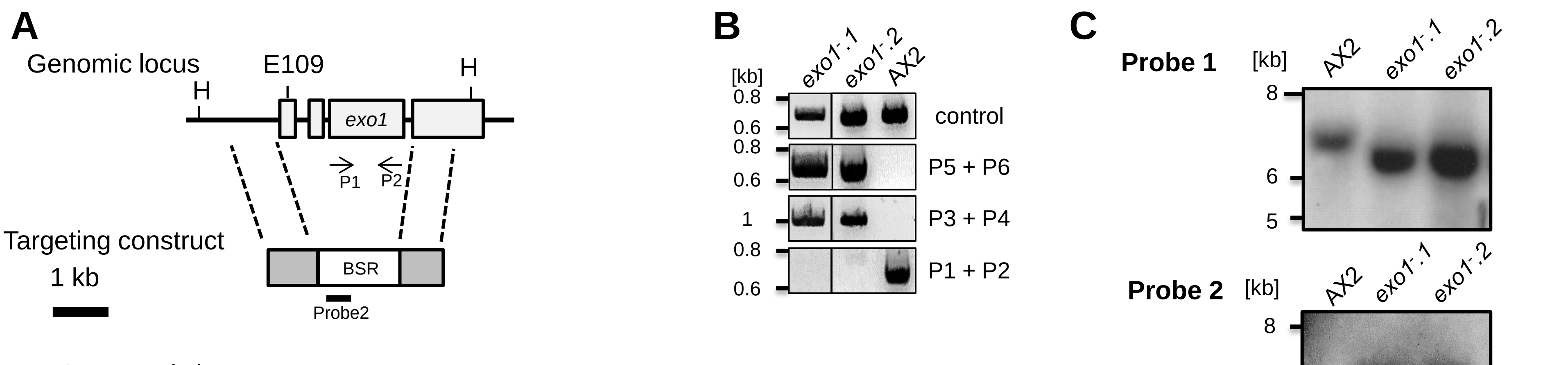

Primers	Expected PCR products (genomic DNA, bp)	
	AX2	*exo1⁻*
control	618	618
P1 + P2	710	N/A
P3 + P4	N/A	1059
P5 + P6	N/A	640

Supplementary Figure S2

A. Strategy for the disruption of the *exo1* genomic locus by targeted homologous recombination. The *exo1* genomic locus is shown in the parental Ax2 cells (top) and the *exo1*$^{-}$ cells (bottom). Homology regions in the targeting construct (middle) are depicted as grey boxes. This strategy disrupts the entire exonuclease activity of *exo1* and leaving 920bp remaining in the final exon (white boxes). P indicated the Primers used for screening by PCR, H indicates restriction enzyme site of HaeIII used for southern blotting, Probe 1 and Probe 2 indicates the location of the probes used for southern blotting. **B**. PCR verification of the *exo1*$^{-}$ strains using the primers at locations indicated in (A). The expected fragment sizes for Ax2 and *exo1*$^{-}$ strains are shown in the table. **C.** Southern Blot verification of the *exo1*$^{-}$ strains. Genomic DNA of Ax2 and *exo1*$^{-}$ strains were digested with HaeIII (H) and using the probes, indicated in (A), results in detection of DNA fragments shown in the table. Probe 1 confirms disruption of the *exo1* genomic locus, whereas blotting with probe 2 binds to blasticidin resistance cassette (BSR) confirming a single integration event had occurred in the disruption strains.

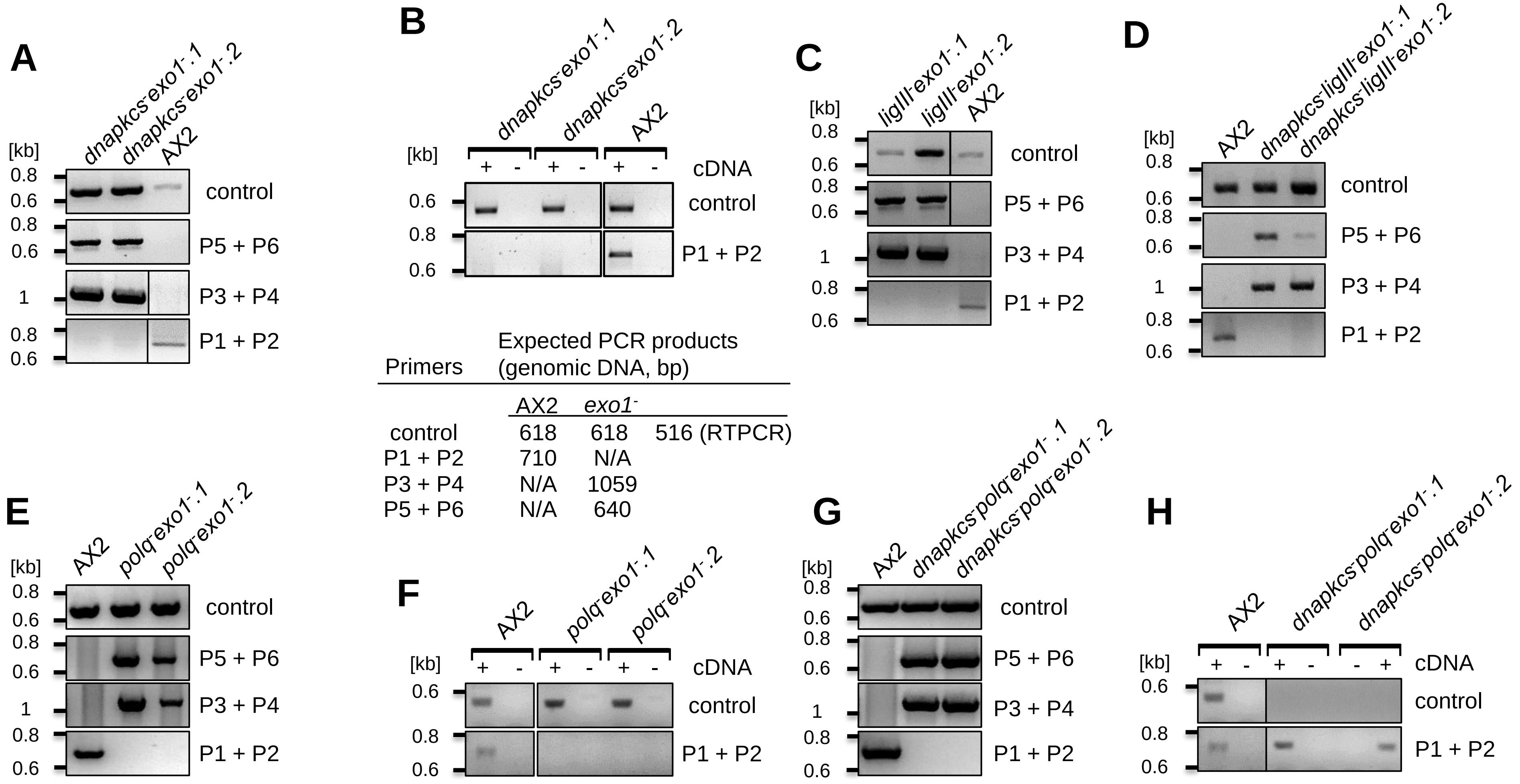

Primers	Expected PCR products (genomic DNA, bp) AX2	*exo1⁻*	
control	618	618	516 (RTPCR)
P1 + P2	710	N/A	
P3 + P4	N/A	1059	
P5 + P6	N/A	640	

Supplementary Figure S3

A. PCR verification of disrupting the *exo1⁻* genomic locus in the *dnapkcs⁻* strain using the primers at locations indicated in (S2A). The expected PCR product sizes are given in the table. **B.** RT-PCR verification of *dnapkcs⁻exo1⁻* using the primers at locations indicated in (S2A). The expected PCR product sizes are given in the table. **C.** PCR verification of disrupting the *exo1⁻* genomic locus in the *ligIII⁻* strain using the primers at locations indicated in (S2A). The expected PCR product sizes are given in the table. **D.** PCR verification of disrupting the *exo1⁻* genomic locus in the *dnapkcs⁻ligIII⁻* strain using the primers at locations indicated in (S2A). The expected PCR product sizes are given in the table. **E.** PCR verification of disrupting the *exo1⁻* genomic locus in the *polq⁻* strain using the primers at locations indicated in (S2A). The expected PCR product sizes are given in the table. **F.** RT-PCR verification of *polq⁻exo1⁻* using the primers at locations indicated in (S2A). The expected PCR product sizes are given in the table. **G.** PCR verification of disrupting the *exo1⁻* genomic locus in the *dnapkcs⁻polq⁻* strain using the primers at locations indicated in (S2A). The expected PCR product sizes are given in the table. **H.** RT-PCR verification of *dnapkcs⁻polq⁻exo1⁻* using the primers at locations indicated in (S2A). The expected PCR product sizes are given in the table.

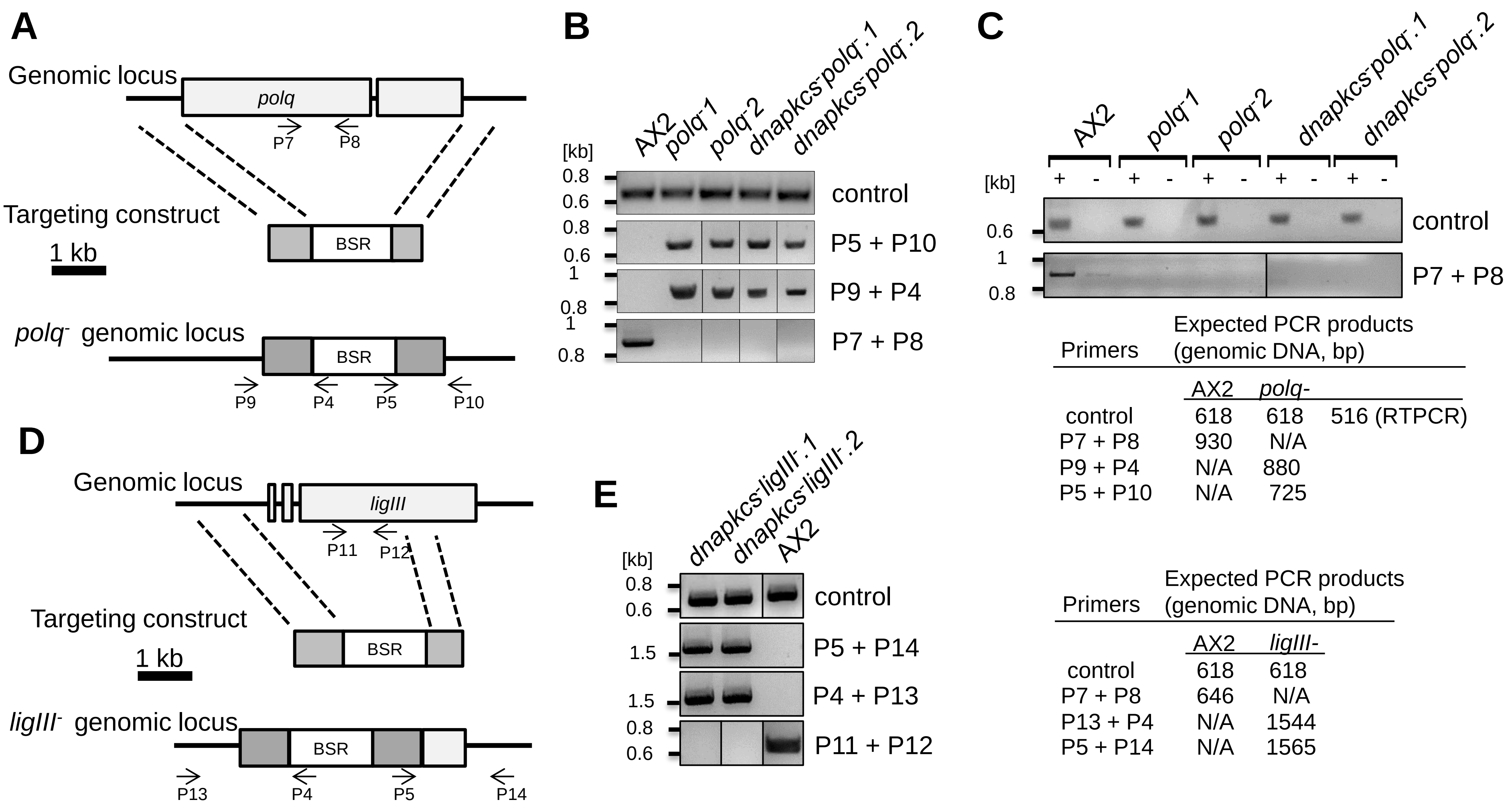

Primers	Expected PCR products (genomic DNA, bp)		
	AX2	*polq-*	
control	618	618	516 (RTPCR)
P7 + P8	930	N/A	
P9 + P4	N/A	880	
P5 + P10	N/A	725	

Primers	Expected PCR products (genomic DNA, bp)	
	AX2	*ligIII-*
control	618	618
P7 + P8	646	N/A
P13 + P4	N/A	1544
P5 + P14	N/A	1565

Supplementary Figure S4

A. Strategy for the disruption of the *polq* genomic locus by targeted homologous recombination. The *polq* genomic locus is illustrated in the AX2 (top) as well as *polq*$^-$ cells (bottom). Homology regions in the targeting construct (middle) are depicted as grey boxes. This strategy disrupts almost the entire *polq* genomic locus, with only the last 800bp remaining. P indicated the Primers used for screening by PCR. **B.** RT-PCR verification of *polq*$^-$ strain and *dnapkcs*$^-$*polq*$^-$ using the primers at locations indicated in (A). The expected PCR product sizes are given in the table **C.** PCR verification of disrupting the *polq*$^-$ genomic locus in the indicated strains using the primers at locations indicated in (A). The expected PCR product sizes are given in the table. **D.** Strategy for the generation of a *dnapkcs*$^-$*ligIII*$^-$ strain by targeted homologous recombination. The *ligIII* genomic locus is depicted in the parental Ax2 cells (top) and the *ligIII*$^-$ cells (bottom). Homology regions in the targeting construct (middle) are depicted as grey boxes. This strategy disrupts most of the *ligIII* genomic locus, with only the last 600bp remaining of the final exon. P indicated the Primers used for screening by PCR. **E.** PCR verification of disrupting the *dnapkcs*$^-$*ligIII*$^-$ genomic locus in the indicated strains using the primers at locations indicated in (D). The expected PCR product sizes are given in the table.

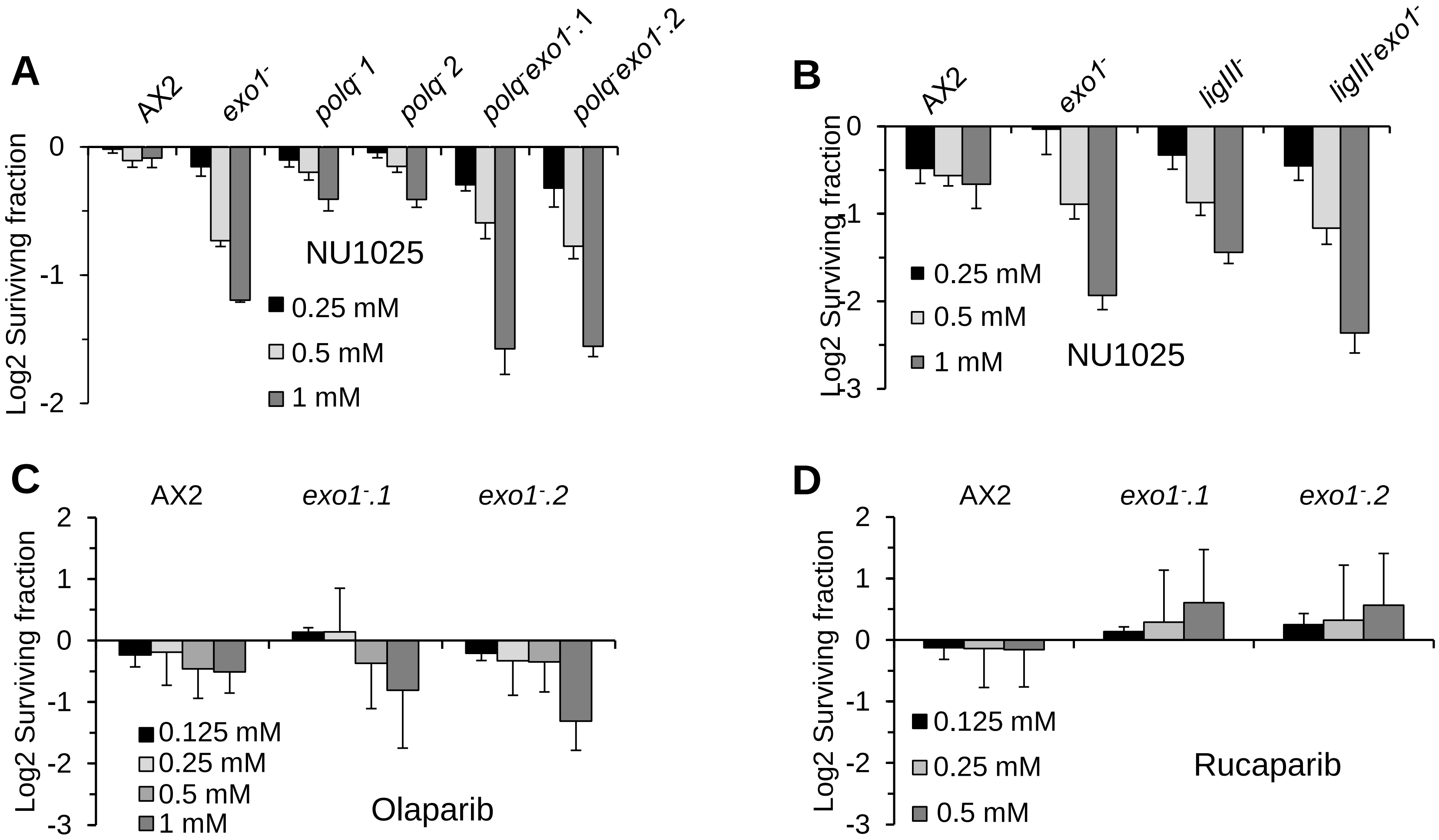

Supplementary Figure S5

A. Ax2, *exo1*-, two independent *polq*-, and two independent *polq*-*exo1*- strains were incubated with the indicated NU1025 concentration or mock treated. Cell viability was assessed after 5 days using *CellTiter-Glo*® (Promega). Cell viability is presented as log2 fold changes between untreated and treated samples. Error Bars represent the SEM of three independent experiments. **B.** Ax2, *exo1*-, *ligIII*- and *ligIII*-*exo1*- strains were incubated with the indicated NU1025 concentration or mock treated. Cell viability was assessed after 5 days using *CellTiter-Glo*® (Promega). Cell viability is presented as log2 fold changes between untreated and treated samples. Error Bars represent the SEM of three independent experiments. **C and D.** Ax2 and two independent *exo1*- strains were incubated with the indicated olaparib (left) or rucaparib (right) concentration or mock treated. Cell viability was assessed after 5 days using *CellTiter-Glo*® (Promega). Cell viability is represented as log2 fold changes between untreated cells and those exposed to ARTi. Error Bars represent the SEM from three independent experiments.
